# Resveratrol: Harnessing Nature's Potential for Chronic Pain Relief

**DOI:** 10.14336/AD.2025.0530

**Published:** 2026-06-22

**Authors:** Hong Wu, Jia-Yi Wu, Shao-Jie Gao, Lin Liu, Xin-Yi Dai, Wen-Lu Song, Long-Qing Zhang, Dai-Qiang Liu, Ying-Xin Tang, Ya-Qun Zhou, Wei Mei

**Affiliations:** ^1^Department of Anesthesiology and Pain Medicine, Hubei Key Laboratory of Geriatric Anesthesia and Perioperative Brain Health, and Wuhan Clinical Research Center of Geriatric Anesthesia, Tongji hospital, Tongji Medical College, Huazhong University of Science and Technology, Wuhan, Hubei, China.; ^2^Department of Neurology, Tongji Hospital, Tongji Medical College, Huazhong University of Science and Technology, Wuhan, Hubei, China.

**Keywords:** Resveratrol, chronic pain, neuroinflammation, oxidative stress, autophagy, 5-HT

## Abstract

Resveratrol, a natural polyphenol with anti-inflammatory, antioxidant, and neuroprotective properties, shows great potential in managing chronic pain. This review explores its analgesic mechanisms, including the inhibition of neuroinflammation, enhancement of antioxidant activity, induction of autophagy, reduction of endoplasmic reticulum stress, modulation of the serotonin system, restoration of gut microbiota homeostasis, regulation of the neuroendocrine system, and promotion of mitochondrial biogenesis. While its analgesic potential is considerable, future research should prioritize enhancing its bioavailability, investigating drug interactions, and confirming long-term safety to develop more effective therapies for chronic pain.

## 1. Introduction

Chronic pain represents a debilitating condition that significantly impairs quality of life, affecting not only physical health but also mental well-being. In response to the growing burden of pain-related disorders, leading medical institutions have established specialized pain management clinics to provide diagnostic and therapeutic services for patients suffering from various chronic pain conditions. Of course, a variety of analgesics are also employed in pain management, including opioids, acetaminophen, and non-steroidal anti-inflammatory drugs (NSAIDs) [1]. Nonetheless, the long-term use of these medications is often limited by adverse effects, highlighting the urgent need for alternative natural therapies to alleviate chronic pain effectively.

Resveratrol, a bioactive phenolic compound renowned for its antioxidant properties, is extensively found in various plant species [2, 3]. The emerging potential therapeutic benefit of resveratrol as an analgesic has garnered increasing attention from researchers [4]. It has been demonstrated that resveratrol is involved in the modulation of various types of chronic pain, encompassing neuropathic, inflammatory, visceral, and bone cancer pain [5-8]. While resveratrol has been highly valued as an effective analgesic against chronic pain, its analgesic action does not arise from a single pathway but through multiple mechanisms. To comprehensively evaluate the role of resveratrol in chronic pain management, we systematically searched the PubMed, Web of Science, Scopus, and Cochrane Library to collect relevant literature up to December 2024, ensuring the inclusion of all important studies related to the analgesic mechanisms of resveratrol. This review assesses the efficacy of resveratrol in treating chronic pain conditions and discusses the underlying mechanisms by which it alleviates pain, thus providing valuable clinical perspectives for the management of chronic pain.

## 2. Main molecular targets of resveratrol

**Figure 1.**
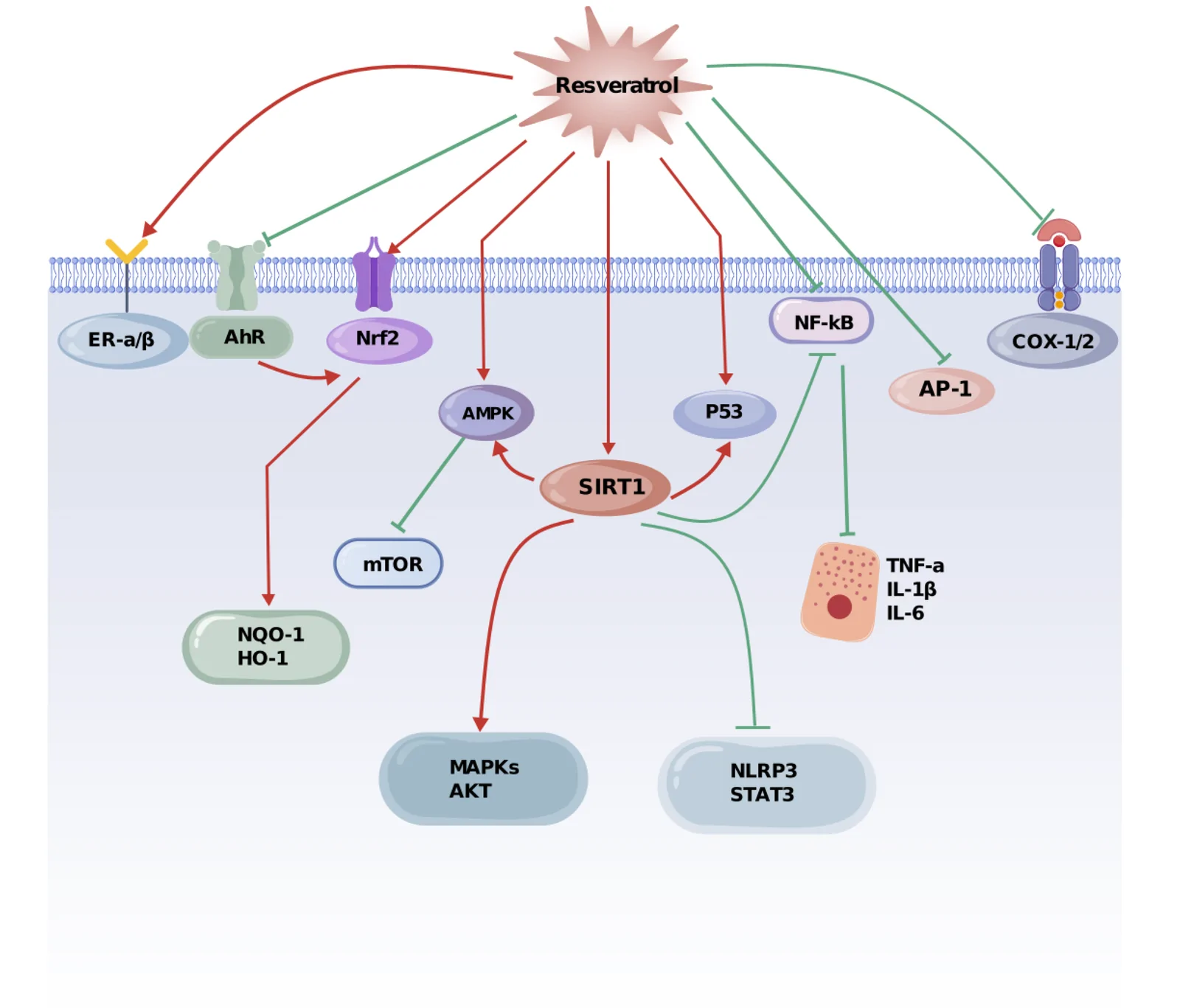
**The main molecular targets of resveratrol**. The ovals represent potential direct targets, and the boxes represent potential indirect targets. These direct targets include AhR (aryl hydrocarbon receptor), Nrf2 (nuclear factor erythroid 2-related factor 2), COX (cyclooxygenase), SIRT1 (silent information regulator 1), AP-1 (activator protein-1), NF-κB (nuclear factor kappa B), AMPK (adenosine monophosphate activated protein kinase), P53(tumor protein 53), and ER (estrogen receptor). These targets play a crucial role in the anti-inflammatory, antioxidant, and analgesic mechanisms of resveratrol. →: promotion; ⊥: inhibition.

Here, we review a few key biological targets of resveratrol, as depicted in Figure 1 (created with BioGDP.com), to reveal the underlying mechanisms of this potential analgesic polyphenol. At the molecular level, aryl hydrocarbon receptor (AhR) is the first target identified. Resveratrol is a potent competitive antagonist of AhR that inhibits the effects related to AhR activation [9]. AhR functions as a ligand-activated transcription factor and plays regulatory roles in various biological functions, including the modulation of inflammation and maintenance of homeostasis [10]. Upon activation, AhR potentiates Nrf2/NAD(P)H quinone oxidoreductase 1 (NQO1) signaling, subsequently decreasing the synthesis of pro-inflammatory cytokines in mouse colonic tissues [11]. For instance, resveratrol has been shown to protect intestinal integrity and mitigate intestinal inflammation and oxidative stress (OS) in diquat-challenged piglets by modulating the AhR/Nrf2 pathway [12]. Nrf2, a transcription factor with anti-inflammatory and antioxidant properties, also plays a crucial role in enzyme biotransformation and lipid/carbohydrate biosynthesis [13]. Notably, resveratrol has demonstrated neuroprotective effects against Parkinson's disease by specifically targeting the Nrf2 signaling pathway [14]. Various studies have evidenced that resveratrol prevents inflammation and diminishes the harmful effects of reactive oxygen species in a wide array of tissues. Moreover, resveratrol augmented Nrf2 protein expression, alongside the activity of the heme oxygenase-1 (HO-1) signaling cascade in newborn rats, thereby alleviating OS and inflammatory responses induced by anoxic environments [15].

Cyclooxygenase (COX) is a key enzyme in prostaglandin synthesis and plays an important role in the regulation of inflammation and pain. Some studies have indicated that resveratrol is capable of downregulating COX-1 and COX-2 expression [16], which are key enzymes in inflammation and pain transmission. By inhibiting the activities of these enzymes, resveratrol reduces inflammation and mitigates chronic pain severity. In addition, as a peroxide-mediated, reversible inhibitor of prostaglandin peroxidase synthase, resveratrol exhibits low inhibitory activities against COX-1 and COX-2 enzymes [17], further attesting to its suitability for application in anti-inflammatory and analgesic therapy.

Of particular interest, resveratrol also acts as an activator of anti-aging enzymes, among which silent information regulator (SIRT1) has been the most highly studied target. SIRT1, an NAD^+^-dependent histone deacetylase, has been associated with both inflammatory responses and neuroprotection [18]. Based on existing evidence, resveratrol could modulate various signaling pathways in a SIRT1-dependent way, such as increasing the phosphorylation of MAPKs, AKT, and AMPK, while inhibiting the activation of the NLRP3 inflammasome, NF-κB, and STAT3. Furthermore, resveratrol reduces β-amyloid levels via the SIRT1-NF-κB-BACE1 signaling pathway [19]. By lowering the Michaelis constant of SIRT1 for its acetylation substrate and NAD^+^, and promoting SIRT1-dependent p53 deacetylation, resveratrol enhances cell survival [20]. The role of p53 in apoptosis has been well-established, wherein it pauses the cell cycle to facilitate repair of cellular damage, including DNA. In the event of widespread DNA damage, p53 initiates apoptotic pathways to inhibit the spread of genetically unstable cells and tumor formation [21]. Resveratrol also enhances the sensitivity of lung cancer cells to the anticancer agent TRAIL by independently inhibiting the Akt/NF-κB signaling pathway through p53 [22]. To ensure adequate levels of ATP in the cell in response to energy and cellular stress, AMPK needs to regulate various catabolic and anabolic signaling pathways [23]. Resveratrol is also the activator of adenylate cyclase and AMPK, which can directly affect AMPK and its related signaling pathways. Depending on the dosage, resveratrol can increase AMPK phosphorylation while simultaneously decreasing mTOR phosphorylation, ultimately leading to the induction of autophagy [24]. Summary, resveratrol, as an inhibitor or activator, has shown a variety of effects, including reducing inflammation-related cell expression, inhibiting cancer cell growth and inducing apoptosis, inhibiting COX-2 expression, and promoting autophagy.

Activator protein-1 (AP-1) and NF-κB are transcription factors evolutionarily conserved across eukaryotic organisms. They act in concert or independently as master regulators of the expression of target genes implicated in a wide array of physiological processes. NF-κB activation can lead to the expression of inflammatory cytokines such as interleukin 1beta (IL-1β), IL-6, IL-10, and tumor necrosis factor (TNF) in lipopolysaccharide-stimulated cells [25]. NF-κB signaling pathway plays an important role in mediating resveratrol's anti-inflammatory effects. It has been demonstrated that resveratrol suppresses toll-like receptor 4 (TLR4) expression and inhibits NF-κB activation in vitro. In addition, in hypoxia/reoxygenation-induced injury, resveratrol suppresses the expression of TNF-α and IL-1β, making it a promising therapeutic agent for preventing inflammation due to such injury [26]. Moreover, resveratrol modulates gene transcription by activating stimulus-responsive protein kinases and AP-1 [27]. Specifically, extracellular signal-regulated protein kinase (ERK) and p38 MAPK have been shown to modulate 12-O-tetradecanoylphorbol-13-acetate (TPA) -induced NF-κB activation. In vivo investigations conducted on mouse skin have demonstrated that p38 MAPK and JNK play pivotal roles in the activation of AP-1 following TPA administration [28]. Currently, NF-κB and AP-1 are regarded as the primary molecular targets for the chemoprophylactic effects of resveratrol. However, the complex and cell-type-specific interactions between these two transcription factors often complicate the understanding of the distinct mechanisms underlying the chemoprophylactic activity of this phytochemical.

Resveratrol, acting as a potent agonist of estrogen receptor (ER)-β, inhibits cellular proliferation and enhance cell survival [29]. These properties may contribute to its anti-inflammatory effects, which are crucial for pain management. By binding to ER-β, resveratrol inhibits cellular proliferation and growth and enhances cell survival through promoting the expression of mitochondrial superoxide dismutase [30]. More recently, studies have shown that resveratrol's function is ER-α-mediated, altering the recruitment of co-regulators associated with ER-α and making ER-α a primary target in regulating the inflammatory response [31]. In brief, resveratrol's major activities involve its anti-inflammatory and antioxidant effects through multiple targets with intricate mechanisms. Some of the targets mentioned in this section also participate in the analgesic mechanisms of resveratrol.

## 3. Potential mechanism of resveratrol's analgesic effect in chronic pain

### 3.1 Inhibition of neuroinflammation

Neuroinflammatory processes are characterized by the activation of resident immune cells, including microglia and astrocytes, as well as infiltration by peripheral immune cells. Pro-inflammatory cytokines and chemokines are produced after the activation of immune cells. Inflammatory mediators like these disrupt the integrity of the blood-brain barrier and hence induce neuronal damage [32]. Current research has identified that such an activation of glial and immune cells results in the escalation of pro-inflammatory mediators, which contributes to a neuroinflammatory milieu. This mechanism is taken to be a fundamental process whereby acute pain initiates an evolution into neuropathic and chronic pain disorders [33,34]. One main way resveratrol reduces pain is by halting inflammatory signal activation and cellular inflammation. It offers symptomatic protection to individuals experiencing pain by downregulating TNF-α, IL-1β, and IL-6, while also modulating the expression of the pro-inflammatory mediators COX-2 and inducible nitric oxide synthase (iNOS) [8, 35-38]. Various molecules involved in the initial immune response and different stages of the inflammatory process are regulated by NF-κB, such as interleukins, tumor necrosis factors, iNOS, COX-2, chemokines, adhesion molecules, and colony-stimulating factors. Resveratrol's protective effect against ischemia-reperfusion-induced vascular peripheral neuropathy is attributed to the inhibition of NF-κB signaling [39]. Furthermore, current understanding indicates that the canonical WNT pathway acts as an anti-inflammatory mechanism, whereas the non-canonical pathway promotes inflammation. Resveratrol modulates the WNT/β-catenin pathway and its associated inflammatory responses, including interleukins, TNF, NF-κB, iNOS, and COX-2, thereby mitigating behavioral changes related to low back pain [37]. Additionally, the JAK2/STAT3 signaling pathway is essential in numerous physiological and pathological processes, including inflammatory responses [40]. As a natural inhibitor of the STAT3 pathway, resveratrol has been shown to effectively alleviate abnormal mechanical pain following spinal cord injury in rats by partially inhibiting the JAK2/STAT3 signaling pathway [38].

Extracellular ATP acts as a signaling molecule in contexts of neuropathic and inflammatory pain, activating ionotropic P2X receptors in primary afferent fibers [41]. Notably, P2X7 receptors are essential for activating afferent nerves and are implicated in inflammation, chronic neuropathic pain, and pain associated with cancer. Resveratrol demonstrates a protective effect against neuropathic pain transmission by interacting with P2X7 and P2X3 receptors [42-44]. Additionally, resveratrol alleviates pain by inhibiting the activity of matrix metalloproteinases (MMPs), such as MMP2, MMP9, and MMP13, which are involved in signal transduction and can degrade and activate various cytokines [35, 45].

Resveratrol is well-recognized as a SIRT1 agonist, exerting a wide range of cellular functions in various experimental models through SIRT1-dependent mechanisms. Specifically, resveratrol enhances pain relief by upregulating SIRT1 expression or improving its enzymatic activity. For instance, Yin et al. reported that resveratrol promotes pain relief in rat models of neuropathic pain by activating SIRT1 in the spinal cord [48]. In the chronic contractile injury (CCI) model, SIRT1 activated by resveratrol suppressed the expression of Nav1.7 in dorsal root ganglia through miR-182, thus exerting a pain-relieving effect in rats [47]. Resveratrol can significantly reverse antinociceptive tolerance by activating spinal cord SIRT1 and inhibiting spinal cord total acetyl-histone H3 (Ac-H3) [48]. This is the first study to highlight the role of spinal cord III histone/protein deacetylases in the analgesic effects of resveratrol within a morphine-tolerant rat model. Importantly, resveratrol's anti-inflammatory effects are not solely due to SIRT1 activation but also involve the concurrent inhibition of various inflammatory factors. In addition, resveratrol has been shown to decrease neuroglial activation and reduce inflammatory hyperalgesia in a SIRT1-dependent manner while also lowering IL-6 and TNF-α expression, demonstrating its extensive influence on pain modulation pathways [49].

Activated microglia, serving as the primary immu.ne cells in the central nervous system (CNS), play a pivotal role in neuroinflammation. Their activation can subsequently induce the activation of astrocytes, thereby alter their functional phenotype and contributing to the process of neuroinflammation [50]. Certain investigations have suggested that pain transmission encompasses not merely nerve cells but also non-neuronal entities, notably glial cells. These glial cells are closely related to clinically relevant pain models. Their activation may cause pain amplification through the induction of neuro-inflammation, which increases susceptibility to pain [51]. Resveratrol suppresses inflammation by inhibiting the activation of glial cells, hence reducing inflammatory pain and bone cancer pain, as well as trigeminal neuralgia; this involves crucial adenosine AMPK activation [6, 52-54]. Furthermore, resveratrol attenuates inflammation via the AMPK/dynamin-related protein 1 (Drp1) signaling cascade, effectively suppressing bone cancer pain in rats [55]. Additionally, the analgesic efficacy of resveratrol in neuropathic pain scenarios is exerted through quenching inflammatory cytokines, thus inhibiting glial cell activation [56].

Neuroinflammation, a critical immune response in the nervous system, is a key driver of chronic pain. It involves the activation of microglia and astrocytes, leading to the release of pro-inflammatory cytokines that exacerbate pain signaling. These inflammatory mediators can cause neuronal injury, which manifests as pain, a cardinal symptom of inflammation [57]. The interrelationship between neuroinflammation and pain unfolds in a cascading manner. In summary, resveratrol possesses the capacity to forestall neuroinflammation at its inception, thereby alleviating pain by preventing glial cell activation and the generation of inflammatory mediators.

### 3.2 Antioxidant effects

OS, characterized by the excessive accumulation of reactive oxygen species (ROS), disrupts the cellular reduction-oxidation (REDOX) balance and contributes to neuronal dysfunction and cell death. This process is a key factor in the development and persistence of chronic pain conditions. The body possesses inherent mechanisms to counteract OS, primarily antioxidants [58]. An imbalance between oxidative processes and antioxidant defenses is associated with various pain-related pathophysiological conditions. Viggiano et al. found increased production of O_2_^-^ in a facial pain model and involvement in pain transmission associated with inflammation [59]. Resveratrol, a potent natural antioxidant, alleviates pain by neutralizing ROS and restoring the cellular redox balance. This mechanism is particularly important in reducing oxidative stress-induced inflammation, a common feature of chronic pain. The primary enzymes of the antioxidant system include superoxide dismutase (SOD), catalase (CAT), and glutathione peroxidase (GSH-Px). It has been found that intraventricular injection of resveratrol in rats with neuropathic pain induced by nerve ligation can normalize CAT and SOD activity, which is interrupted after neuropathic pain and reduce pain [60]. Furthermore, one study assessed the function of resveratrol in the system of early and late inflammatory pain defense, especially the TNFR1/extracellular ERK signaling pathway and levels of ROS. It was revealed that resveratrol reduced inflammatory pain hypersensitivity by downregulating the activation of ERK and modulating the differential regulation of ROS and antioxidant enzymes [61].

Although OS and neuroinflammation are distinct pathological processes, they are closely interconnected and can influence each other. In particular, OS can modify the inflammatory response, thereby contributing to the complex interplay between these two processes. Realizing this relation, Recalde et al. demonstrated that resveratrol enhanced the levels of antioxidant mediators like Nrf2, NQO-1, and HO-1; suppressed pro-inflammatory parameters like NF-κB, TNF-α; and reduced mechanical and thermal pain development caused by oxaliplatin (OXA) [62]. The PI3K/Akt pathway holds a crucial position in regulating nociceptive signals and is integral to microglial signaling pathways. Resveratrol also manifested neuroprotection through the PI3K/Akt pathway, under which the neuronal cells are safeguarded against OS, hence highly reducing neuropathic pain induced by paclitaxel [63]. Excessive reactive nitrogen radicals (RNS) can also cause OS, including nitric oxide (NO), nitrogen dioxide (NO_2_), and nitrite peroxide. Intrathecal administration of resveratrol alleviates abnormal tactile pain in neuropathic rats and reverses the decrease in NO synthase activity and neuronal NOS expression caused by spinal nerve ligation [64]. As signaling intermediates, ROS and RNS facilitate the release of pro-inflammatory cytokines and activate the MAPK pathway through NF-κB, thus regulating neuroinflammation and exacerbating pain [65]. Therefore, resveratrol's antioxidant and anti-inflammatory effects often complement each other.

Furthermore, it is possible that transient receptor potential (TRP) channels will be a target in the treatment of chronic pain. Diabetic peripheral neuropathy is linked to excess intracellular ROS and mitochondria-derived ROS (mtSOX) [66]. In fact, the activation of TRPV4 initiates an intracellular cascade resulting in neuronal injury and OS, thereby promoting peripheral neuropathy with increased mtSOX levels [67]. TRPV4 stimulation augmented the sensitivity of diabetic neuropathic pain and cytoplasmic Ca^2+^ inflow, and injections of resveratrol reversed these changes [68]. Perhaps resveratrol modulates neuropathic pain by inhibiting TRPV4 channels as a result of its antioxidant effects.

### 3.3 Activation of autophagy

Disrupted autophagy might cause a broad spectrum of pain manifestations, suggesting that this cellular process could be very important in pain pathways and thus a viable target for therapeutic interventions against pain [69]. Autophagy is a self-degradation process that regulates the degradation of proteins and organelles, thereby enhancing cell viability under stress [70]. The study indicated that enhancing autophagy can effectively reduce neuropathic pain by decreasing the levels of pro-inflammatory cytokines that significantly contribute to neuropathic pain development [71]. Additionally, resveratrol has emerged as one of the promising chemo-preventive and chemotherapeutic agents that could protect normal cells while initiating apoptosis in cancerous cells through the process of autophagy induction [72]. Resveratrol also exhibits analgesic properties by modulating autophagy. It specifically stimulates the AMPK-SIRT1-autophagy signaling cascade by elevating the phosphorylation states of SIRT1, AMPK, and LC3-II, thereby alleviating pain in rodent models of bone cancer [73]. Additionally, resveratrol can also relieve neuropathic pain by regulating the TREM2-autophagy axis and inhibiting microglia-mediated neuro-inflammation in rats with residual nerve injury [74]. Fan et al. demonstrated that resveratrol enhances mitochondrial membrane potential, represses P62 and Pink1 expression, upregulates LC3B-II, Parkin, and TOMM20, and promotes mitochondrial autophagy, leading to the relief of gout arthritis [75]. Based on above studies, the analgesic effects of resveratrol are further enhanced through autophagy, a process governed by a diverse array of regulatory pathways.

### 3.4 Inhibition of endoplasmic reticulum stress (ERS)

The ER represents a specific organelle responsible for protein synthesis and correct folding. The unfolded protein response (UPR), which is activated in response to ERS, functions as a protective mechanism primarily intended to rejuvenate ER function. Nevertheless, persistent ERS can ultimately lead to cellular malfunction and the induction of apoptosis [76]. Resveratrol exerts beneficial effects by modulating both aspects of ERS: it attenuates excessive UPR activation, thereby delaying or preventing apoptosis and tissue damage, while also reducing the protein-folding burden on the ER to enhance cell survival [77]. Additionally, ERS-mediated adaptive responses have been shown to play a critical role in pain modulation and may represent a novel therapeutic strategy for various neuropathic conditions [78]. Furthermore, resveratrol has been shown to provide protective benefits to many organs by regulating ER stress, including the liver, heart, and kidneys [79-81]. Studies focusing on pain alleviation have also been conducted. By means of an ischemic reperfusion (IR) -induced peripheral vascular neuropathy (VPN) model, Pan et al. observed that resveratrol significantly reduced NF-κB and ERS sensor-associated proteins. Their observation showed that resveratrol may weaken IR-induced VPN by inhibition of ERS and modulation of NF-κB-mediated neuroinflammation [39]. It is evident that ERS is a key factor in pain development, and resveratrol's role in pain relief is closely linked to its anti-inflammatory properties.

### 3.5 Activation of serotonergic system

Serotonin (5-HT) acts as a primary neurotransmitter, influencing various receptor subtypes in both neuronal and non-neuronal systems to mediate its effects [82]. The serotonergic system is diffusely and highly distributed in the CNS and participates in a wide array of processes, including pain modulation, analgesia, sleep-wake regulation, and autonomic functions [83]. As a biogenic amine, 5-HT directly influences pain perception by activating several 5-HT receptor subtypes. Among these, 5-HT7 receptor has garnered significant attention due to its substantial role in modulating both inflammatory and neuropathic pain paradigms. Consequently, 5-HT 's action on this receptor is crucial in the complex neural mechanisms underlying pain sensation and perception. In addition, the serotonergic system plays an important role in the regulation of injury perception, mainly by regulating descending pain circuits associated with neuroplastic changes in different brain regions [84]. Prior research has shown that systemic administration of selective 5-HT7 receptor agonists leads to a significant reduction in mechanical and thermal hypersensitivity responses by activating 5-HT7 receptor. Notably, co-administration of selective 5-HT7 receptor antagonists reverses this reduction, further corroborating the involvement of 5-HT7 receptor subtype in pain regulation [85]. New potential uses of 5-HT7 receptor agonists in the treatment of neuropathic pain are also indicated. The monoaminergic system is not only heavily involved in descending pain regulation but also accountable for the effects of resveratrol on chronic stress-induced damage [86, 87]. Other studies have suggested that the neurological role of resveratrol in antidepressants is mainly through the regulation of 5-HT [88]. Studies have shown that when central 5-HT levels are chemically depleted, the analgesic effects of resveratrol on mice with neuropathic pain disappear. In contrast, co-administration of resveratrol with a 5-HT precursor enhances its pain-relieving properties. Moreover, the highly spinal cord-localized 5-HT7 receptor has been found to be critical for the analgesic action of resveratrol against heat stimulation [89]. These results confirm that the serotonergic system, especially the implication of 5-HT7 receptor, constitutes part of the mechanism underlying the action of resveratrol in neuropathic pain management.

### 3.6 Restoration of gut microbiota

Microbial mediators play a key role in regulating neuroinflammation in the CNS. These mediators interact with key components of the neuroinflammatory cascade, such as the activation of microglia, immune cells, and the blood-brain barrier [90]. As a result, they significantly influence the induction and maintenance of central sensitization, which is a fundamental mechanism underlying the onset and persistence of chronic pain. A growing body of evidence indicates that alterations in gut microbiota composition are associated with various types of chronic pain, including visceral, nociceptive, headache-related, and neuropathic pain. For instance, fecal microbiota from mice with visceral allergies, compared to healthy controls, were transferred into antibiotic-treated rats, resulting in long-term visceral allergies accompanied by changes in intestinal levels of short-chain fatty acids [91]. Various signaling molecules within the gut microbiota, such as metabolic products, neurotransmitters, and neuromodulators, through specific receptors significantly influence peripheral and central sensitization, hence contributing to the development of chronic pain [90]. Studies have demonstrated that resveratrol can alter the composition of gut microbiota in obese mice, which importantly influences glucose homeostasis [92]. Thus, resveratrol might have the potential to be used for the regulation of gut microbiota. Additionally, research has demonstrated that resveratrol effectively suppresses complete Freund's adjuvant (CFA)-induced temporomandibular joint inflammation by reversing short-chain fatty acids and associated gut microbiota, restoring blood-brain barrier integrity, inhibiting microglia activation, and decreasing TNF-α release within the spinal cord [93]. These observations imply that resveratrol may ameliorate inflammatory pain by normalizing gut microbiota, preserving blood-brain barrier integrity, and consequently modulating microglial activation and the secretion of pro-inflammatory cytokines. Therefore, targeting neuroinflammatory processes modulated by gut microbiota-derived mediators presents a novel and promising therapeutic avenue for treating chronic pain.

### 3.7 Regulation of neuroendocrine system

Neuroendocrine system changes, both centrally and peripherally, play a crucial role in pain modulation. Glucocorticoid negative feedback mechanisms are often disrupted in traumatic stress patients, particularly affecting the functionality of the limbic hypothalamus-pituitary-adrenal (HPA) axis [94]. Trans-resveratrol can reduce pain sensitization linked to disabilities resulting from post-traumatic stress by precisely modulating the activity of the HPA axis via its action on glucocorticoid receptor (GR). This modulation triggers the activation of downstream neuroprotective signaling pathways, including PKA and phosphorylated cAMP response element-binding protein. Besides, it upregulates the expression of brain-derived neurotrophic factor (BDNF), thereby contributing to the attenuation of pain sensitization linked to post-traumatic stress-related disabilities [95]. Such aspects give evidence for the therapeutic utility of trans-resveratrol in pain conditions associated with post-traumatic stress disorders. BDNF is the most abundant neurotrophic factor, essential for neuronal survival and neurotransmitter release. The production of BDNF depends on Ca^2+^, while increased levels of BDNF decrease pain sensitivity. On the other hand, increased intracellular calcium levels can inhibit BDNF gene expression and might interfere with the analgesic effect of resveratrol [96]. In summary, other specific analgesic effects of resveratrol may be achieved by regulating the neuroendocrine system.

### 3.8 Excitation of mitochondrial biogenesis

Resveratrol can stimulate mitochondrial biogenesis and enhance bioenergetics associated with mitochondria [97]. It has been reported to induce mitochondrial biogenesis in different tissues, especially nervous tissue, and lately there is a tide of interest in its potential neurotherapeutic use [68]. Moreover, related studies have suggested that resveratrol may reduce mitochondrial damage and dysfunction under neuropathic pain conditions [63, 98]. Indeed, impairment of mitochondrial autophagy may also cause mitochondrial accumulation, leading to further impairment of mitochondrial biogenesis and function, thereby aggravating mitochondrial dysfunction [99]. Resveratrol may also mitigate pain in gouty arthritis through inhibition of mitochondrial autophagy [75]. Hence, mitochondrial biogenesis may be a mechanism by which resveratrol exerts its analgesic action.

**Table1:** Summary of preclinical evidence of the therapeutic potential of resveratrol in chronic pain.

Model	Treatment Strategy	Effects	Mechanisms	Ref.
CCI-induced neuropathic pain rats	RSV (300 μg, i.t.) was administered once per day 7 days after CCI for 4 successive days.	PWT↑ PWL↑	SIRT1, miR-182↑ Nav1.7↓	[47]
TCI-induced bone cancer pain rats	RSV (30, 100 and 300 mg/kg, p.o. or 10 μg/20μl and 30 μg/20μl, i.t.) was administered on day 14 after TCI. Resveratrol (300 mg/kg, p.o. or 30 μg/20μl, i.t.) was administered on days 14, 15, 16, 17 and 18 after TCI.	PWT↑	p-AMPK↑ glial activation↓ IL-1β and TNF-α↓ p-NR1, p-PKCγ, and p-MAPKs ↓ p-Akt and p-mTOR↓	[6]
TNBS-induced visceral pain mice	RSV (10 µL 4.5 mM or 10 µL 45 mM, i.t.) was administered from day 4 to 7 post TNBS.	VPT↑	GFAP, TRAF6↓ pNF-κB, TNF-α and IL-1β↓	[7]
CFA-induced inflammatory pain rats	RSV (20mg/kg, i.p.) was administered 20 minutes before CFA injection.	PWL↑	COX-2 and iNOS↓	[8]
STZ-induced diabetic peripheral neuropathy mice	10% RSV (10 ml/kg, i.g.) was administered once a day until the 12th week after STZ injection 2 weeks.	PWT↑ PWL↑	Nrf2↑ NF-κB ↓	[107]
Carrageenan-induced inflammatory pain rats	RSV (2mg/kg, i.p.) was administered one hour before carrageenan injection.	PWT↑	PGE2↓ COX-2↓	[108]
CCI-induced neuropathic pain mice	RSV (30 mg/kg, p.o.) was administered twice per day for three weeks after CCI 2 weeks.	PWT→ PWL↑	5-HT7↑ MAO activity↓	[89]
STZ-induced diabetic peripheral neuropathy mice	RSV (5, 10 and 20 mg/kg/d, p.o.) was administered at 4 to 8 weeks after STZ injection.	PWL↑	Nitrite↓ TNF-α↓	[109]
pSNL-induced neuropathic pain rats	RSV (200mg/kg, i.p.) was administered into rats once daily for 21 days starting on the day of pSNL surgery.	PWT↑ PWL↑	P2X3↓ p-ERK↓	[42]
SNL-induced neuropathic pain rats	RSV (40 μg/5 μL, i.v.) was administered on day 1 and day 6 after SNL surgery.	PWL↑	NCV↑ CAT and SOD activities↑	[60]
CCI-induced trigeminal neuralgia mice	RSV (200, 400 and 600 mg/kg, p.o.) was administered 30 minutes before CCI surgery.	PWT↑	MMP-9, MMP-2↓ TNF-α, IL-1β↓ p-NR1, p-PKCγ↓ SOCS3↑	[45]
Monosodium iodoacetate-induced osteoarthritic pain rats	RSV (5 and 10 mg/kg/d, p.o.) was administered from day 0 to 14 daily after MIA injection.	PWT↑ PWL↑	IL-1β, IL-6, IL-10 and TNF-α↓ MMP-13↓ iNOS and COX-2↓	[35]
CFA-induced inflammatory pain rats	Resveratrol (20 mg/kg, i.p.) was administered immediately after CFA injection.	PWL↑	antioxidant defense system p-ERK, ERK↓ TNFR1→	[61]
IR-induced vasculitic peripheral neuropathy rats	Resveratrol (20 and 40 mg/kg, i.p.) was administered 10 minutes before IR surgery.	PWT↑ PWL↑	p-IκB and p-NFκB-p65↓ PERK, IRE1 and ATF6↓	[39]
CFA-induced temporomandibular joint inflammation mice	RSV (40 mg/kg or 80 mg/kg, i.p.) was administered once a day for consecutive 4 days starting from 1 h post-CFA.	PWT↑	SCFAs↑ gut microbiota	[93]
Carcinoma cells inoculation -induced bone cancer pain rats	RSV (30 µg/d and 300 µg/d, i.t.) was administered at 1 day before to 7 days after carcinoma cells inoculation. RSV (30 µg/d and 300 µg/d, i.t.) was administered post-inoculation days 14-16.	PWT↑	CX3CR1↓ glial activation↓	[52]
TCI-induced bone cancer pain rats	RSV (0.3, 1, and 3 mg/kg, i.p.) was administered on post-operative Day 21.	PWT↑	ASIC3↓ p-AMPK, LC3-II, SIRT1↑	[73]
LPS-induced inflammatory pain mice	RSV (5, 10 or 20 mg/kg, i.p.) was administered 30 minutes before LPS injection.	Number of rithes↓ Tail-flick latency↑	IL-6, TNF-α↓ SIRT1↑ glial activation↓	[49]
Morphine-induced morphine-tolerant rats	RSV (30 µg, i.t.) was administered on days 7-13 after morphine injection.	PWL↑	SIRT1↑ Ac-H3↓	[48]
CCI-induced neuropathic pain mice	RSV (5, 10, 20 and 40 mg/kg, i.p.) was administered from day 7 to day 14 after CCI.	PWT↑ PWL↑	TNF-α, IL-1β, IL-6↓ IL-10↑	[36]
CFA-induced rheumatoid arthritis mice	RSV (15 or 25 mg/kg, i.p.) was administered once daily starting on day 22 and continuing for two weeks after CFA injection.	PWT↑	TNF-α, IL-1β↓ PADI4, COX-2↓ NET release↓ NF-κB↓	[110]
CCI-induced neuropathic pain rats	RSV (200 mg/kg, i.p.) was administered once daily for 14 consecutive days after CCI surgery.	PWT↑ PWL↑	IL-1RA, IL-1R2, IL-4Rα↑	[111]
OXA-induced peripheral neuropathic pain rats	RSV (between 6,28 and 7,81 mg/kg/day, p.o.) was administered starting 4 days before the chemotherapy cycle and thereafter, until the end of the experiment (1 or 3 weeks after initiating OXA cycle).	PWT↑ PWL↑	NF-κB, TNF-α, ATF3↓ Nrf2, NQO-1, HO-1 and SIRT1↑ TBARS↑	[62]
CCI-induced neuropathic pain rats	RSV (300 µg/d, i.t.) was administered from 4 to 7 days after CCI surgery.	PWT↑ PWL↑	SIRT1↑ Ac-H3↓	[46]
CFA-induced intravertebral disc degeneration rats	RSV (3mg/kg, i.g.) was administered daily by gavage until the seventh day after CFA injection.	PWT↑ PWL↑	WNT3a, FZ8, β-catenin↓ IL-18, TNF-α and IL-1β↓ IkB -α↑ NF-kB↓ iNOS, COX-2↓ GSH, SOD and CAT↑	[37]
Paclitaxel-induced neuropathic pain rats	RES (TCI) was prepared in 5% sodium carboxymethylcellulose to a concentration of 8 mg/mL and injected i.p. at 40 mg/mL on seven alternate days after Paclitaxel injection.	PWT↑ PWL↑	PI3K, p-Akt, SIRT1 and PGC1α↑ IL-10↑ IL-1β↓ SOD↑ MDA↓ mitochondrial damage↓	[63]
SNI-induced neuropathic pain rats	RSV (300 µg/d, i.t.) was administered 2 consecutive days from 6 to 7 d after SNI surgery.	PWT↑	TREM2-mediated autophagy TNF-α, IL-1β, IL-6↓ IBa1, TREM2↓	[74]
Morphine-induced morphine-tolerant mice	RSV (40, 80 or 160 mg/kg, i.p.) was administered 15 min before morphine injection.	Antinociceptive effect↑	IBa1↓ AMPK↑	[53]
Morphine-induced morphine-tolerant rats	RSV (7.5, 15, 30 or 60 µg, i.t,) was administered after morphine infusion for 5 days.	Antinociceptive effect↑	PSD-95, NR1 and NR2B↓ TNF-α, IL-1β, IL-6↓ glial activation↓	[56]
TCI-induced bone cancer pain rats	RSV (1 mg/kg, i.t.) was administered after TCI.	PWT↑	AMPK and p-AMPK↑ Drp1 GTPase activity↓ Mn-SOD activity↓	[55]
CCI-induced trigeminal neuralgia mice	RSV (100, 200 and 400 mg/kg, p.o.) was administered after CCI surgery 14 days.	PWT↑	AMPK activation↑ glial activation↓ IL-1β and TNF-α↓ p-NR1 and p-PKCγ↓	[54]
Thoracic (T10) spinal cord contusion injury rats	Intrathecal administration with Res (300 μg/10 μl, i.t.) was performed once a day for 7 days after spinal cord contusion injury operation.	PWT↑	p-JAK2 and p-STAT3↓ TNF-α, IL-1β and IL-6↓	[38]
gp120-treated rats	RES (30 mg/kg, i.p.) was administered at 24 h after surgery for 14 days.	PWT↑	P2X7↓ TNF-α, IL-1β↓ IL-10↑ p-ERK1/2 and ERK1/2↓	[43]
STZ-induced diabetic peripheral neuropathy mice	RSV (25 mg/kg, i.p.) was administered for 3 weeks following a single intraperitoneal STZ injection.	PWT↑ PWL↑	TRPV4 activation↓ Oxidative Stress and Mitochondrial Dysfunction	[68]
spinal nerve ligation-induced tactile allodynia rats	RSV (300µg, i.t.) was administered on day 15 after spinal nerve ligation surgery.	PWT↑	NOS↑	[64]
CCI-induced neuropathic pain rats	RSV (25 mg/kg, i.p.) was administered for 14 days after CCI surgery.	PWT↑ PWL↑	P2X7↓ TNF-α and IL-6↓ GFAP↓	[44]
Single-prolonged stress-induced neuropathic pain mice	RSV (10, 20 and 40 mg/kg, i.p.) was administered for 21 days after single-prolonged stress.	PWT↑ Response score of cold allodynia↓	GR ↓ BDNF↑	[95]
/	RSV (10, 20 and 40 mg/kg, p.o) was administered 1 h before testing.	PWL↑	pCaMKII↓ BDNF↑	[96]
MSU-Induced arthritis rats, MSU-induced peritonitis mice, inflammatory models of mouse bone marrow-derived macrophage	RSV (10, 25 and 50 mg/kg, i.g.) was administered for 7 consecutive days. RSV (15 mg/kg, i.p.) was administered on day 2.	/	IL-1β, IL-18 and Caspase-1↓ NLRP3, P62 and Pink1↓ LC3B-II, TOMM20↑ mitophagy↑	[75]

**Abbreviations:** PWL: paw withdrawal latency; PWT: paw withdrawal threshold; RSV: resveratrol; i.t.: Intrathecal; i.g.: Intragastric; i.p.: Intraperitoneal; p.o.: Per os; i.v.: Intravenous; CCI: chronic constriction injury; SIRT1: silent information regulator 1; TCI: tumor cell implantation; AMPK: adenosine monophosphate activated protein kinase; IL-1β/6/10/18/1RA/4Rα/1R2: interleukin 1beta/6/10/18/1RA/4Rα/1R2; TNF-α: tumor necrosis factor α; NR1: N-methyl-d-aspartate receptor 1; PKCγ: protein kinase C γ; MAPK: mitogen-activated protein kinase; Akt: protein kinase B; mTOR: mammalian target of rapamycin; TNBS: 2,4,6-Trinitrobenzene sulfonic acid; GFAP: glial fibrillary acidic protein; VPT: visceral pain threshold; TRAF6: TNF receptor associated factor 6; NF-κB: nuclear factor-κB; CFA: complete Freund’s adjuvant: iNOS: inducible nitric oxide synthase; COX-2: cyclooxygenase 2; Nrf2: nuclear erythroid 2-related factor 2; STZ: streptozotocin; PGE2: prostaglandin-E2; 5-HT: 5-hydroxytryptamine (serotonin); MAO: monoamine oxidase; pSNL: partial sciatic nerve ligation; P2X3/7: purinergic receptor P2X 3/7; ERK: extracellular signal-regulated protein kinase; NCV: nerve conduction velocity; CAT: catalase; SOD: superoxide dismutase; Mn-SOD: manganese superoxide dismutase; MMP-9/2/13: matrix metalloproteinase-9/2/13; SOCS3: suppressor of cytokine signaling 3; TNFR1: TNF-α receptor 1; IR: ischemia-reperfusion; ATF6/3: activating transcription factor 6/3; PERK: protein kinase RNA-like endoplasmic reticulum kinase; IRE1: inositol-requiring enzyme 1; SCFAs: short-chain fatty acids; OXA: oxaliplatin; CX3CR1: CX3C chemokine receptor 1; ASIC3: acid-sensing ion channels 3; LC3: light chain 3; LPS: lipopolysaccharide; Ac-H3: acetyl-histone H3; NETs: neutrophil extracellular traps; PADI4: peptidyl arginine deiminase 4; HO-1: heme-oxygenase 1; NQO-1: NAD(P)H quinone oxidoreductase; TBARS: thio-barbituric acid reactive substances; WNT3a: wingless-type MMTV integration site family-3a; FZ8: frizzled class receptor 8; GSH: pyrimidodiazepine synthase; PI3K: phosphoinositide 3-kinase; PGC1α: peroxisome proliferative activated receptor, gamma, coactivator 1 alpha; MDA: malondialdehyde; SNI: spared nerve injury; TREM2: triggering receptor expressed on myeloid cells 2; IBa1: induction of brown adipocytes 1; PSD-95: postsynaptic density 95; Drp1: dynamin-related protein 1; JAK2: janus kinase 2; STAT3: signal transducer and activator of transcription 3; gp120: HIV envelope glycoprotein 120; TRPV4: transient receptor potential vanilloid 4; NOS : nitric oxide synthase; GR: glucocorticoid receptor; BDNF: brain-derived neurotrophic factor; CaMKII: calmodulin-dependent protein kinase II; MSU: monosodium urate; NLRP3: NLR family pyrin domain containing 3; TOMM20: translocase of outer mitochondrial membrane 20.

## 4. Concluding remarks and future perspective

**Figure 2.**
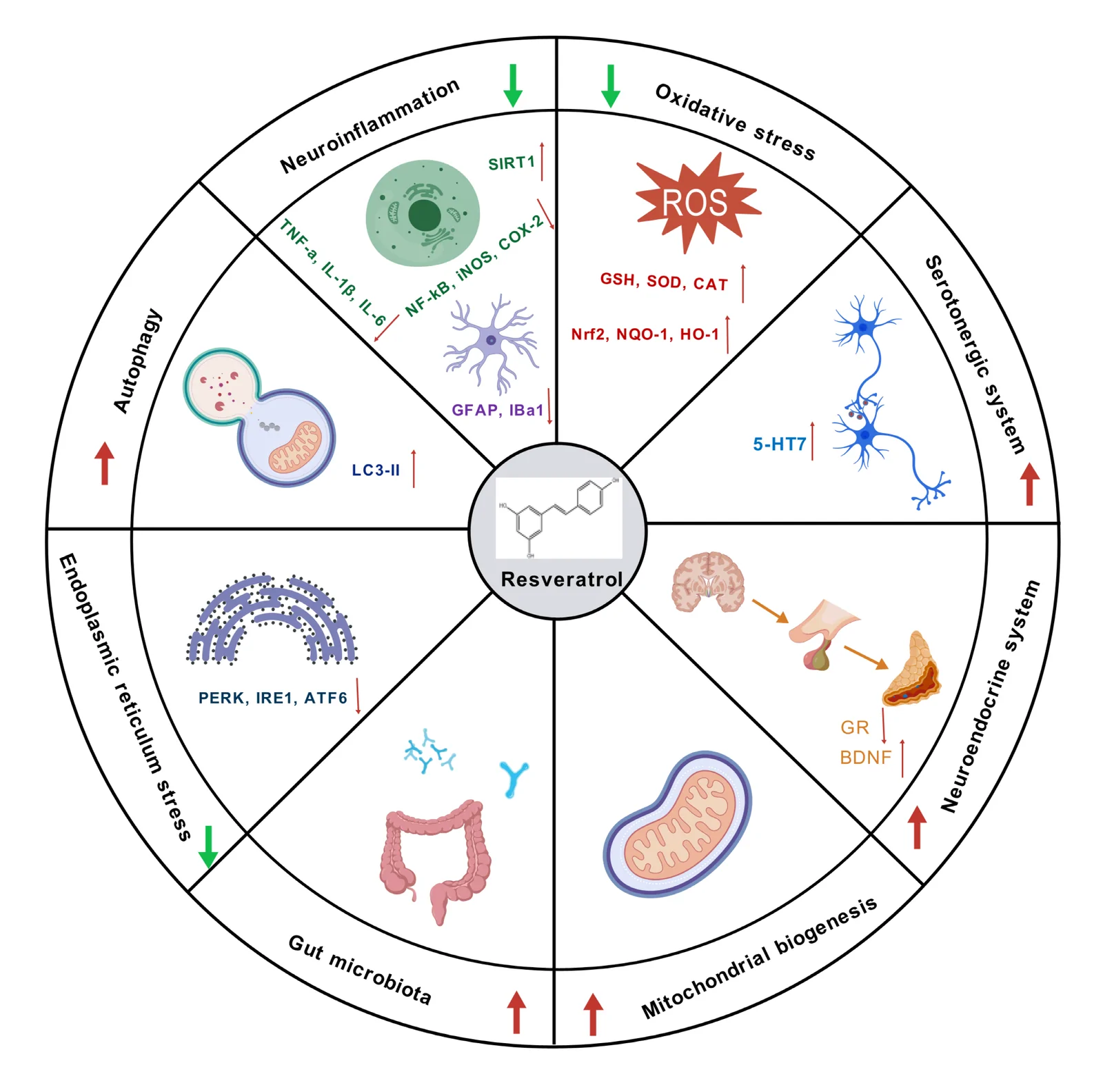
**The underlying mechanisms responsible for the analgesic action of resveratrol in preclinical investigations**. The analgesic properties of resveratrol are manifested through eight core aspects, including the inhibition of neuroinflammation, antioxidant effects, activation of autophagy, inhibition of endoplasmic reticulum stress, activation of the serotonergic system, restoration of the gut microbiota, regulation of the neuroendocrine system, and excitation of mitochondrial biogenesis. “↑” indicates increase, activation, or promotion; “↓” indicates reduction or inhibition.

This article presents various mechanisms through which resveratrol alleviates chronic pain, summarized in Table 1. The analgesic properties of resveratrol are manifested through eight core aspects, as shown in Figure 2 (created with BioGDP.com), including neuroinflammation inhibition, antioxidant activity, autophagy induction, attenuation of ERS, regulation of the serotonergic system, gut microbiota restoration, modulation of the neuroendocrine system, and induction of mitochondrial biogenesis. These mechanisms collectively highlight the potential of resveratrol as a novel therapeutic approach for chronic pain management. Despite its potent analgesic effects, several key questions regarding resveratrol's mechanisms remain to be further investigated and validated.

Although the present study provides useful data on the potential application of resveratrol for chronic pain treatment, it also has some methodological limitations. Firstly, there are significant differences in the doses and routes of resveratrol administration across different studies, which may affect the comparability of the results and the feasibility of clinical application. Furthermore, most studies rely on animal models, which vary significantly in the simulation of human chronic pain complexity. For example, it is impossible to fully replicate the pathophysiological mechanisms of human chronic pain in animals, particularly in terms of neuroinflammation and OS. Choosing appropriate animal models plays a critical role in rendering research outcomes precise and translatable. These limitations would mean that future studies would have to be more cautious in standardizing doses and selecting animal models to ensure study reliability and translational utility of study results.

Evidence now indicates significant sex differences in the incidence and progression of chronic pain, which are related to fluctuating levels of sex hormones, endogenous analgesic mechanisms, structural and functional brain differences, and psychosocial factors related to gender roles. These differences can significantly impact the efficacy and safety of therapeutic interventions. While most of the evidence provided here is taken from experiments using male rodents, this gender bias restricts our knowledge regarding how resveratrol may have differential effects in male versus female subjects. Future preclinical research must systematically incorporate female subjects to have a complete picture of these gender differences. In addition, clinical trials should also consider sex as a biological variable to further improve the identification of the therapeutic potential of resveratrol in male and female patients. This approach will help in developing more effective and personalized treatment strategies for chronic pain management.

Moreover, numerous studies have confirmed the effectiveness of resveratrol for pain relief, especially in neuropathic and inflammatory pains. Among the targets identified, SIRT1 is the most extensively studied, although its role is primarily associated with neuroinflammation. Therefore, further research is needed to elucidate these mechanisms and identify additional molecular targets responsible for resveratrol's effects on different pain types, which could inform personalized treatment strategies for pain management.

Besides, the increasing number of clinical trials has greatly increased social awareness and recognition of resveratrol. Clinical research has demonstrated that resveratrol can improve bone mineral density in postmenopausal women and offers protective effects for patients suffering from cardiovascular and metabolic diseases [100-102]. These findings support the view that resveratrol is a safe and adequate option, but its pharmacokinetic profile and further toxicity concerning pain management need to be comprehensively studied in clinical trials before general clinical application.

Currently, derivatives of resveratrol have also garnered the attention of researchers. The advantages and disadvantages of resveratrol and its derivatives can vary significantly. The advantages of resveratrol lie in its clear bioactivity, diverse mechanisms of action, and abundant natural sources that are easily accessible. However, resveratrol also has some limitations, such as low bioavailability, which may restrict its effective concentration and therapeutic effects in the body [103]; at high doses, it may interact with certain medications, and its therapeutic efficacy can vary among individuals [104]. On the contrary, resveratrol analogs are more advantageous in terms of bioavailability and stability, with some having stronger antioxidant and anti-inflammatory properties and less cytotoxicity [105, 106]. They also have disadvantages, however, such as comparatively fewer studies, unconfirmed efficacy and safety, and potentially higher production costs due to complicated synthetic pathways. Further, their natural availability and abundance are not as favorable as that of resveratrol. Therefore, future studies on the therapy of chronic pain must fully consider the advantages and disadvantages of resveratrol and its analogs to optimize their utilization and obtain maximum benefits.

In summary, resveratrol functions as a natural therapeutic agent, which shows great potential for developing new analgesic medications and provides essential clinical value for treating chronic pain. Though it has been shown to exert analgesic effects in various models of chronic pain, most current research is confined to the experimental level only, and clinical safety and efficacy are yet to be explored in depth. As a choice for an alternative analgesic, additional clinical studies must be conducted to investigate the practical use of resveratrol in the treatment of chronic pain. Future research must aim at increasing the bioavailability of resveratrol, ascertaining drug interactions, and confirming safety in prolonged use, to ultimately offer more effective treatment for chronic pain patients.
